# Emerging Trends in Global Cancer Epidemiology

**DOI:** 10.3390/cancers17091483

**Published:** 2025-04-28

**Authors:** Syed Ahsan Raza

**Affiliations:** Section of Epidemiology and Population Sciences, Department of Medicine, Baylor College of Medicine, Houston, TX 77546, USA; s.ahsanraza@gmail.com

Cancer continues to be one of the most pressing global public health challenges, with an increasing number of new cases and deaths each year. As the global burden of cancer grows, so too does the need for robust epidemiological research that illuminates patterns in incidence, mortality, prevalence, and survival across diverse populations and regions (Contribution 1). This Special Issue, entitled ‘Emerging Trends in Global Cancer Epidemiology’, brings together insightful research that collectively reflects the dynamic landscape of cancer epidemiology worldwide. These contributions offer novel perspectives on cancer trends, highlight innovative methods—including the use of big data, genomics, and machine learning—and explore the influence of environmental, lifestyle, and social determinants on cancer outcomes. From analyses of population-level disparities to evaluations of prevention strategies, each study in this issue adds a unique piece to the global cancer puzzle. The full collection of articles featured in this Special Issue can be accessed at the following link: https://www.mdpi.com/journal/cancers/special_issues/N946HDMKIS.

In one study in this Special Issue, Aldhaleei et al. explore the rising burden of early-onset colorectal cancer (EO-CRC) among individuals aged 20 to 44 in the United States over a three-decade period (Contribution 2). Using data from the Global Burden of Disease Study 2021 and an age–period–cohort (APC) modeling approach, the authors revealed a 49% increase in EO-CRC cases from 1990 to 2021, with age-standardized incidence rates rising by 34%. These findings emphasize the complex interplay of age, generational cohort, and period in shaping EO-CRC trends and highlighting the urgency of developing targeted prevention strategies to address this growing incidence in younger populations.

Beltran-Ontiveros et al. present a comprehensive analysis of cancer trends in Mexico over a 30-year period, also drawing on data from the Global Burden of Disease Study 2019 (Contribution 3). From an epidemiological standpoint, this study is a critical reminder of how national-level data can offer valuable insights into shifting cancer dynamics. It also reflects the growing complexity of cancer epidemiology in middle-income countries like Mexico, where demographic transitions, urbanization, and health system challenges may be driving these divergent trends. This research underscores the importance of cancer control strategies that are sensitive to both sex-specific trends and the heterogeneous nature of cancer burden across different cancer types. As countries like Mexico move toward universal health coverage, this kind of granular evidence will be instrumental in shaping equitable cancer prevention and control policies.

Abboud et al. carried out analyses to investigate an emerging and somewhat underrecognized trend in the U.S.—the rising incidence of rectal neuroendocrine tumors (RNETs) in younger adults under the age of 55 (Contribution 4). Using data from the United States Cancer Statistics (USCS) database from 2001 to 2020, the authors conducted a detailed analysis stratified by age, sex, race, and tumor stage. This paper raises important questions about what might be fueling the rise of RNETs in younger demographics. The age-specific divergence in incidence trends is compelling, especially given the growing body of literature pointing to earlier onset patterns across several gastrointestinal malignancies. The fact that the increase is largely driven by early-stage diagnoses suggests that enhanced detection may play a role—but it also hints at possible shifts in risk exposures, lifestyle factors, or even gut microbiome changes in younger generations. This study underscores the need for more targeted research into the etiology and risk profile of RNETs in young adults, and it serves as a call to action for clinicians to revisit current screening thresholds and symptom awareness strategies for this tumor type.

Interesting research carried out by Hussan et al. explores the relationship between obesity, bariatric surgery (BRS), and the development of colorectal polyps—a precursor to colorectal cancer using a large, propensity-matched U.S. nationwide cohort from the 2012–2020 MarketScan Insurance Claims Research Database (Contribution 5). From a clinical epidemiological perspective, this study offers important insights into the preventive potential of bariatric surgery beyond metabolic improvements. It challenges us to think of obesity not just as a risk factor for colorectal cancer, but as a modifiable exposure with early pathological consequences that may be mitigated with timely intervention. The finding of the absence of a significant change in polyp rates pre- vs. post-BRS is particularly thought-provoking—it may suggest that while bariatric surgery halts further risk accumulation, it does not necessarily reverse established polyp pathology. This highlights the need for continued colorectal surveillance, even after weight loss interventions and calls for more research into the biological mechanisms linking obesity, metabolic dysfunction, and early neoplastic changes in the colon.

A population-based study in Eastern Sicily, was carried out to explore cancer burden among migrant populations in a group often underrepresented in epidemiological research (Contribution 6). Using data from the regional cancer registry spanning 2004 to 2019, the study assessed proportionate morbidity ratios (PMRs) and odds ratios (ORs) for various cancers in migrants compared to non-migrants. This study provides an important lens into the intersection of migration and cancer epidemiology. The analysis speaks to the need for culturally appropriate cancer prevention strategies. The markedly higher odds of cervical cancer highlight gaps in HPV vaccination and screening among migrant women, while the elevated lung cancer risk suggests environmental or occupational exposures that warrant closer scrutiny.

Marino et al. delve into the critical yet often overlooked dimension of patient-centered communication (PCC) in intercultural cancer care settings in Italy (Contribution 7). Using the ONCode coding system, the researchers analyzed 42 video-recorded oncology consultations involving both Italian and foreign patients to assess how communication dynamics vary by patient background, type of visit (first vs. follow-up), and presence of companions. Interestingly, quantitative analyses revealed no significant differences in PCC quality between Italian and foreign patients. However, qualitative findings uncovered meaningful distinctions, particularly in the nature of interruptions during encounters with foreign patients, which tended to be more frequent and potentially disruptive. The study emphasizes that while language competence is essential, it should not be the sole indicator of communication effectiveness in multicultural medical settings. This research can be seen as a compelling argument for deeper structural sensitivity in care delivery. In a world of increasingly diverse patient populations, achieving true patient-centricity demands more than protocols; it requires active, empathetic listening and a commitment to minimizing avoidable power imbalances in clinical encounters.

In England, Smith et al. address a critical issue in cancer health disparities by examining how diagnostic pathways mediate the impact of comorbidity on survival among patients with diffuse large B-cell lymphoma (DLBCL) and follicular lymphoma (FL) (Contribution 8). This paper unpacks a subtle but powerful mechanism through which structural inequities manifest in clinical outcomes. From an epidemiological standpoint, this is impactful because it highlights a modifiable point in the cancer care continuum, i.e., diagnostic timeliness, that could yield substantial survival gains if addressed effectively. The mediation analysis strengthens the case for interventions that prioritize early, elective cancer detection in patients with complex health profiles. This evidence should inform not just public health programs, but also clinical training and system-level reforms that aim to anticipate diagnostic risk in vulnerable populations.

The fact that rural residents in developed nations face barriers akin to those in low- and middle-income countries such as delayed diagnosis, limited specialist care, and lower health literacy speaks volumes about the persistent structural gaps in healthcare systems. The study by Ramamurthy et al. from Australia is both insightful and concerning (Contribution 9). They conducted a scoping review to examine how rurality influences oral cancer trends across OECD member countries. Drawing from 18 studies selected from an initial pool of over 1100, the review highlights a troubling pattern: despite residing in high-income countries, individuals in rural and remote areas experience disproportionately higher incidence and poorer outcomes for oral and oropharyngeal cancers. The review points to rising rates of tobacco and alcohol use, low awareness of HPV-related cancer risks, and limited access to advanced cancer care as key drivers of this disparity. The burden is especially high among older adults in rural regions of the United States, Australia, Canada, and several European nations emphasizing that geography remains a powerful determinant of cancer inequity, even in well-resourced settings.

Drăgan and Drăgan from Romania provide a comprehensive narrative review on the assessment of venous thromboembolism (VTE) risk in ambulatory cancer patients, an area often under-addressed in clinical practice (Contribution 10). While VTE is a well-recognized complication in hospitalized and perioperative cancer settings with clear guidelines supporting thromboprophylaxis, the same clarity does not extend to ambulatory patients. The review examines existing tools like the Khorana score, highlighting its limitations, and explores emerging risk stratification models, including those incorporating biomarkers, genetic profiles, clinical parameters, and even machine learning approaches. The authors also discuss the potential role of imaging and biomolecular screening in improving risk prediction and guiding individualized patient care.

Finally, Gupta et al. present a novel population-based study exploring the relationship between environmental temperature and survival outcomes in gastroesophageal cancers (GECs) across the United States (Contribution 11). Using data from over 17,000 esophageal and more than 20,000 gastric cancer patients in the SEER database (1996–2015), combined with county-level temperature data from the National Centers for Environmental Information, their study found that warmer average annual temperatures (AAT) were significantly associated with improved overall survival (OS) and disease-specific survival (DSS). Specifically, patients living in regions with an AAT above 53.5 °F experienced an 11–13% improvement in survival outcomes for esophageal cancer and 13–14% for gastric cancer. Moreover, survival improved incrementally by 3–4% with every 5 °F increase in temperature. These associations held true across histological subtypes and were robust to adjustments for key demographic and clinical variables. While the biological plausibility draws on preclinical evidence that cold stress impairs antitumor immunity, this is one of the first large-scale studies to demonstrate a temperature–survival relationship in human cancer populations (Contribution 11). It introduces an environmental and potentially modifiable factor into the complex equation of cancer prognosis. While the retrospective design and potential for confounding must be acknowledged, the implications are far-reaching. These findings encourage us to consider the broader ecological context in which patients live and receive care. They also spark intriguing questions: Could thermal interventions, such as maintaining thermoneutrality or avoiding cold exposure, complement existing treatment strategies? How might these results influence cancer survivorship planning, especially for vulnerable populations in colder regions? This study opens the door for a new interdisciplinary dialogue between oncology, climate science, and environmental health, one that we are only just beginning to understand.

Collectively, the studies featured in this Special Issue weave a rich and diverse tapestry of emerging trends in global cancer epidemiology. They underscore how cancer risk, burden and outcomes are shaped not only by biological and clinical factors, but also by geography, social determinants, lifestyle, environment, and access to care. From rising early-onset cancers in younger populations to disparities rooted in migration, rurality, comorbidity, and even environmental temperature, these studies challenge us to think beyond traditional risk factors and to adopt a more holistic approach in cancer research and control. As cancer epidemiologists, clinicians, and public health practitioners, we must remain agile and innovative in our methods, while grounded in the realities of patients’ lived experiences. Ultimately, advancing the science of cancer in a truly global context requires embracing complexity, fostering collaboration across disciplines, and translating evidence into cancer prevention and care strategies for all populations.

